# Pulcherrimin formation controls growth arrest of the *Bacillus subtilis* biofilm

**DOI:** 10.1073/pnas.1903982116

**Published:** 2019-06-19

**Authors:** Sofia Arnaouteli, D. A. Matoz-Fernandez, Michael Porter, Margarita Kalamara, James Abbott, Cait E. MacPhee, Fordyce A. Davidson, Nicola R. Stanley-Wall

**Affiliations:** ^a^Division of Molecular Microbiology, School of Life Sciences, University of Dundee, DD1 5EH Dundee, United Kingdom;; ^b^Data Analysis Group, Division of Computational Biology, School of Life Sciences, University of Dundee, DD1 5EH Dundee, United Kingdom;; ^c^School of Physics, University of Edinburgh, EH9 3JZ Edinburgh, United Kingdom;; ^d^Division of Mathematics, School of Science and Engineering, University of Dundee, DD1 4HN Dundee, United Kingdom

**Keywords:** *Bacillus subtilis*, growth arrest, biofilm, pulcherrimin

## Abstract

Biofilm formation by *Bacillus subtilis* is a communal process that culminates in the formation of architecturally complex multicellular communities. Here we reveal that the transition of the biofilm into a nonexpanding phase constitutes a distinct step in the process of biofilm development. Using genetic analysis we show that *B. subtilis* strains lacking the ability to synthesize pulcherriminic acid form biofilms that sustain the expansion phase, thereby linking pulcherriminic acid to growth arrest. However, production of pulcherriminic acid is not sufficient to block expansion of the biofilm. It needs to be secreted into the extracellular environment where it chelates Fe^3+^ from the growth medium in a nonenzymatic reaction. Utilizing mathematical modeling and a series of experimental methodologies we show that when the level of freely available iron in the environment drops below a critical threshold, expansion of the biofilm stops. Bioinformatics analysis allows us to identify the genes required for pulcherriminic acid synthesis in other Firmicutes but the patchwork presence both within and across closely related species suggests loss of these genes through multiple independent recombination events. The seemingly counterintuitive self-restriction of growth led us to explore if there were any benefits associated with pulcherriminic acid production. We identified that pulcherriminic acid producers can prevent invasion by neighboring communities through the generation of an “iron-free” zone, thereby addressing the paradox of pulcherriminic acid production by *B. subtilis*.

## Significance

Understanding the processes that underpin the mechanism of biofilm formation, dispersal, and inhibition is critical to allow exploitation and to understand how microbes thrive in the environment. Here, we reveal that the formation of an extracellular iron chelate restricts the expansion of a biofilm. The countering benefit to self-restriction of growth is protection of an environmental niche. These findings highlight the complex options and outcomes that bacteria need to balance to modulate their local environment to maximize colonization, and therefore survival.

Biofilm formation is a survival strategy used by microorganisms to overcome adverse conditions. It is stimulated by diverse environmental signals that, for example, direct the production of the biofilm matrix (1). The extracellular polysaccharides, adhesins, and matrix proteins that are synthesized by the bacterial population surround and attach to the cells (2). The biofilm matrix provides structure to the 3D community and also fulfills many other functions (3) including the sequestration of water and nutrients (4), driving expansion across surfaces, conferring resistance to phage (5), and neutralizing aminoglycoside antibiotics (6).

*Bacillus subtilis* is a gram-positive, spore-forming bacterium which is ubiquitous in the soil and rhizosphere environment. *B. subtilis* biofilms are of agricultural importance as they are linked with plant growth promotion and the provision of protection from pathogens when formed on plant root systems (7, 8). The mature *B. subtilis* biofilm manifests in vitro as an architecturally complex community (9) that is resistant to gas penetration (10) and exhibits extreme hydrophobicity (10, 11). The extracellular biofilm matrix needed for the assembly of the community comprises stable fibers formed by the secreted protein TasA (12–14), an exopolysaccharide (9), and a protein with dual functions in biofilm architecture and hydrophobicity called BslA (15–17).

The process of *B. subtilis* biofilm formation, in the laboratory, begins with the deposition of “founder” cells (18). These cells differentiate to undertake distinct roles (19–21). For example a subpopulation of these cells transcribes the operons needed for biofilm matrix synthesis (22). Over time, the initial founding cell population divides and expands across the surface in an extracellular matrix-dependent manner (18, 23, 24). However, after a period of 2 to 3 d, expansion of the biofilm stops. Here we find that days after the biofilm has stopped expanding a proportion of metabolically active cells remain in the community. Thus, growth arrest is not due to sporulation of the entire population. Rather, we reveal that it is a distinct stage of biofilm formation by *B. subtilis.* Using a combination of genetics and mathematical modeling we connect synthesis of the extracellular iron chelator pulcherriminic acid, and the subsequent deposition of the iron chelate pulcherrimin, to the arrest of biofilm expansion. We determine that in the absence of pulcherriminic acid production, *B. subtilis* forms “ever-expanding” biofilms. We uncover that pulcherriminic acid manipulates the microenvironment of the biofilm through depletion of iron in the resultant pulcherrimin deposit. Complete depletion of iron in the surrounding environment allows *B. subtilis* to defend its niche from neighboring bacteria, whereas a partial depletion in high-iron conditions allows *B. subtilis* to colonize a surface and gain access to nutrients. Taken together these findings highlight a route by which a bacterial biofilm can optimize survival within a changing environment.

## Results

### Growth Arrest Is a Distinct Stage of Biofilm Formation.

**Fig. 1.**
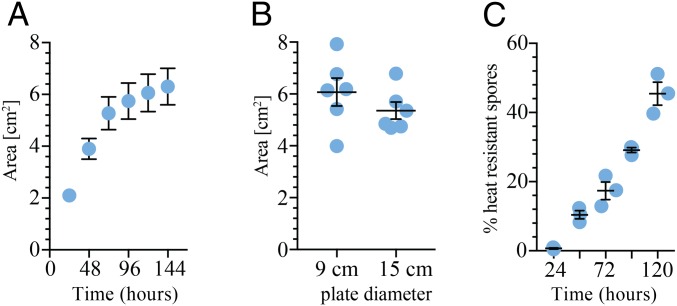
Growth arrest is a distinct stage of biofilm formation. (*A*) The footprint occupied by the NCIB 3610 biofilm was measured over time. The average of three biological repeats is presented; the error bars are the SEM. (*B*) The footprint of the NCIB 3610 biofilm was measured after 120-h growth on agar plates with different diameters. (*C*) The number of heat-resistant spores formed by NCIB 3610 was determined at the time points indicated. In all cases the biofilms were grown at 30 °C on MSgg agar on plates with a diameter of 9 cm unless otherwise stated. For *B* and *C* the bar represents the average of the biological repeats that are presented as individual points and the error bars are the SEM.

*B. subtilis* strain NCIB 3610 forms highly structured hydrophobic biofilms at the air–agar interface. We have noted that mature biofilms reach a finite size (Fig. 1*A*), where the footprint occupied by the community is largely independent of the area of nutrients provided. For example, the average footprints of 5-d-old NCIB 3610 biofilms were ∼5.4 cm^2^ and ∼6.1 cm^2^ when formed on nutrient surfaces with areas ∼176 cm^2^ and ∼63.5 cm^2^, respectively (Fig. 1*B* and *SI Appendix*, Fig. S1*A*). It is known that the genetic background of the bacterium (9) and the environmental conditions used for growth (18, 25) influence the area occupied by the biofilm. For example, using a softer agar (*SI Appendix*, Fig. S1*B*) or depositing the equivalent number of initial cells in a larger volume (*SI Appendix*, Fig. S1*C*) increases the size of the biofilm footprint. However, regardless of experimental variations and subtle differences in the biofilm morphology, the area colonized by the NCIB 3610 biofilm plateaus, despite the apparent availability of nutrients in the surrounding unoccupied surface.

*B. subtilis* is an endospore-forming bacterium and the number of spores increases in the biofilm over time (21, 22). Therefore, a simple hypothesis to explain the arrest in biofilm expansion is that all of the cells in the biofilm have formed spores by 72 h [the point at which the biofilm stops rapidly expanding (Fig. 1*A*)], which would render the cells in the biofilm metabolically inactive. However, we found that while the number of heat-resistant spores within the wild-type biofilm gradually increased over time, even at 120 h the percentage of spores did not reach 100% of the total biofilm cell population (Fig. 1*C*). This leads to the conclusion that a population of cells in the wild-type 120-h-old biofilm remain metabolically active and therefore biofilm expansion must be restricted by some other process.

### Strains Lacking Pulcherriminic Acid Synthesis Form “Ever-Expanding” Biofilms.

**Fig. 2.**
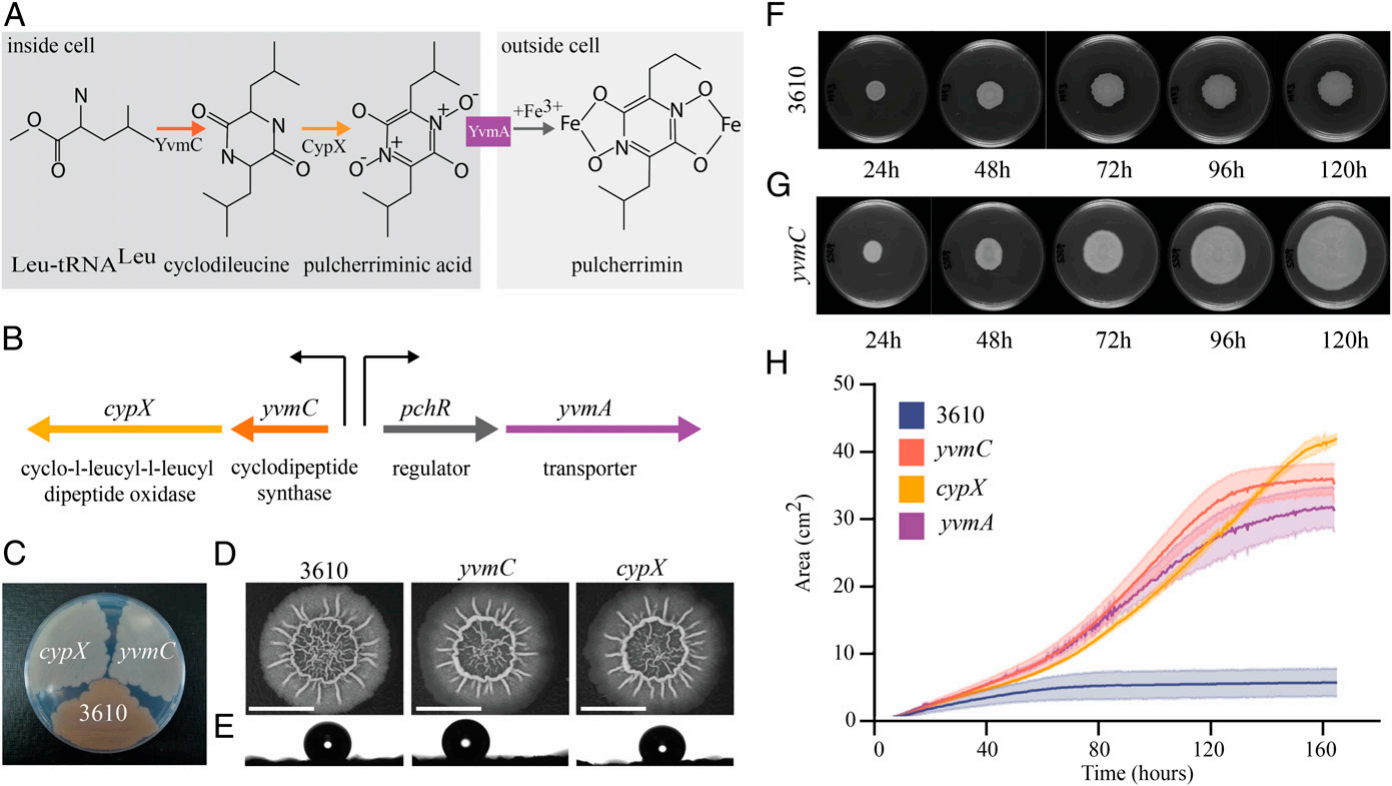
Pulcherrimin production restricts biofilm expansion. (*A*) Schematic depicting the steps involved in the formation of pulcherrimin. (*B*) Genomic organization of the pulcherriminic acid biosynthesis cluster; the raised arrows represent promoters. (*C*) NCIB 3610 and the *cypX* (NRS5532) and *yvmC* (NRS5533) deletion strains after growth on MSgg agar for 24 h at 37 °C. (*D*) Image of biofilms formed by the strains detailed in *C*. (Scale bars, 1 cm.) (*E*) A 5-μL water droplet placed on the biofilms depicted in *D* reveals hydrophobicity. For *D* and *E* the biofilms were grown on MSgg agar for 48 h at 30 °C. (*F* and *G*) Representative images from a time course of biofilm formation showing the area covered by the NCIB 3610 (*F*) and the *yvmC* mutant (*G*). (*H*) Biofilms formed by strains NCIB 3610, *yvmC* (NRS5533), *cypX* (NRS5532), and *yvmA* (NRS6248) were imaged every 20 min for 160 h. The area occupied on the 9-cm-diameter Petri dish was calculated and plotted. The solid lines represent the average of three biological repeats and the shaded area the SEM. The samples in *F*–*H* were grown at 30 °C on MSgg agar containing 50 μM FeCl_3_.

As the mature biofilm contains a significant proportion of cells that are not spores, the arrest in biofilm expansion cannot be due to metabolic inactivity. Therefore, we explored other mechanisms. In biofilms formed by other species of bacteria, pigment production has been linked with biofilm morphology, cell activity, pathogenesis, and survival (26). The *B. subtilis* biofilm produces a secreted, brown pigment that is found in the agar underneath and surrounding the biomass (*SI Appendix*, Fig. S1*A*). We therefore explored if there was a link between production of the pigment and the arrest of biofilm expansion. Pulcherrimin is the only pigment known to be made by *B. subtilis* (27) and is produced when YvmC and CypX convert two tRNA-leucine molecules to pulcherriminic acid. Pulcherriminic acid is secreted from the cell where it binds Fe^3+^ in a nonenzymatic reaction (Fig. 2*A*) (28). The respective genes, *yvmC* and *cypX* (28), are colocated on the genome with the coding regions for a transcriptional regulator (PchR) (29) and a putative membrane-bound transporter, YvmA (30) (Fig. 2*B*).

By introducing mutations in *yvmC* and *cypX* into the parental NCIB 3610 strain, we confirmed that the brown pigment was no longer produced (Fig. 2*C*). We therefore concluded that the pigment produced by the NCIB 3610 biofilm was pulcherrimin. We went on to examine if the *yvmC* and *cypX* deletions impacted biofilm formation. For the first 48 h of growth, the phenotypes of the wild-type and deletion mutant biofilms were virtually indistinguishable with respect to gross architecture (Fig. 2*D*) and hydrophobicity (Fig. 2*E*). In contrast, at later time points, an obvious phenotypic difference manifested. As mentioned earlier, the area occupied by the NCIB 3610 biofilm stops increasing (Fig. 2 *F* and *H*). In the absence of pulcherrimin formation, the biofilms formed by *yvmC* and *cypX* deletion strains continued to expand over time, almost occupying the entire substratum provided (Fig. 2 *G* and *H* and *SI Appendix*, Fig. S2*A*). The ability to keep colonizing the surface was not due to a second site mutation in the genome as the *yvmC* and *cypX* deletion strains could be genetically complemented by provision of the *cypX* and *yvmC* genes under the control of their native promoter, at the ectopic *amyE* locus (*SI Appendix*, Fig. S2 *A* and *B*). These experiments reveal that production of pulcherriminic acid restricts the expansion of the *B. subtilis* biofilm community.

### Radial Expansion of the Pulcherrimin-Deficient Strains.

**Fig. 3.**
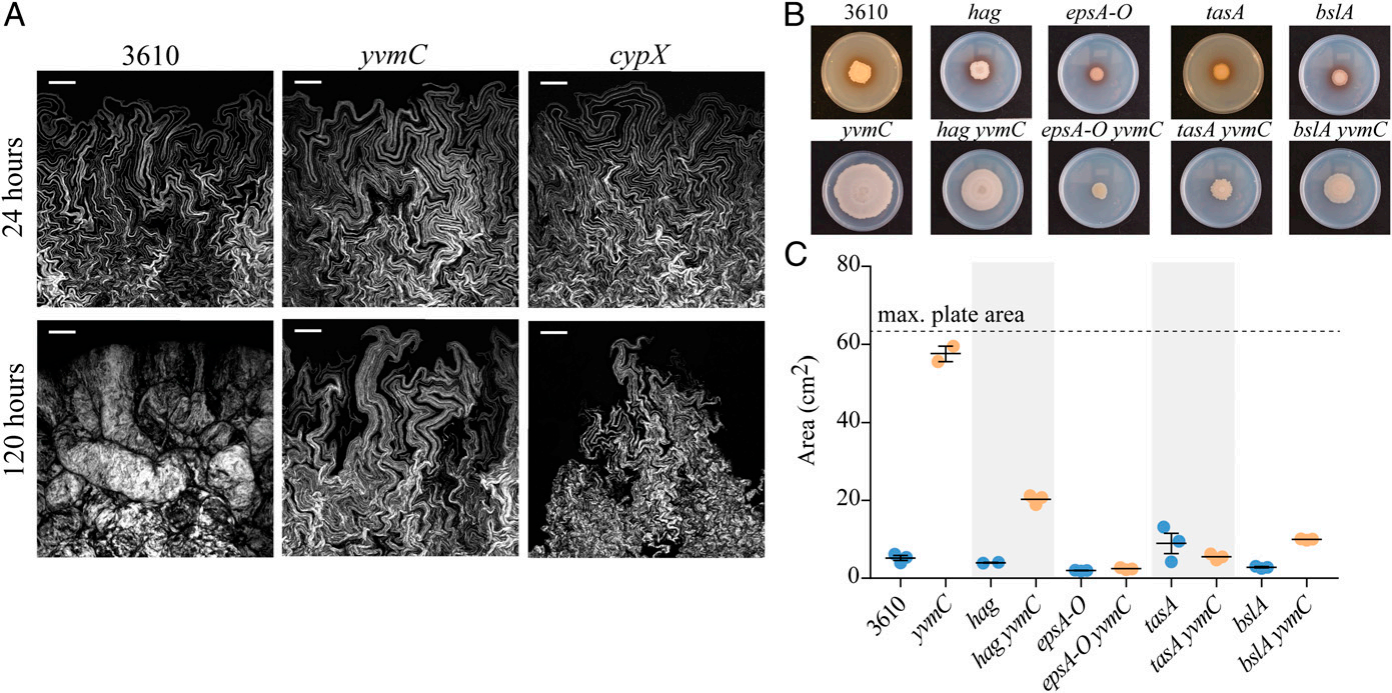
Biofilm expansion in the absence of pulcherriminic acid production. (*A*) Confocal microscopy of the biofilm edge at 24 and 120 h for NCIB 3610 and the *cypX* (NRS5532) and *yvmC* (NRS5533) deletion strains. In each case the strains were mixed with an isogenic variant carrying a constitutively expressed copy of *gfp* (*Materials and Methods* and *SI Appendix*, Table S1). This allowed detection of a fraction of the cells in the biomass by confocal microscopy. The images shown are projections of the acquired z-stacks. In each case the outer edge of the biofilm is on the left-hand side. (Scale bars, 100 μm.) (*B*) Biofilms formed by strains NCIB 3610, *hag* (DS1677), *epsA-O* (NRS2450), *tasA* (NRS5267), *bslA* (NRS2097), *yvmC* (NRS5533), *yvmC hag* (NRS5560), *yvmC epsA-O* (NRS6269), *yvmC tasA* (NRS6276), and *yvmC bslA* (NRS6281) were imaged after 120 h. (*C*) The area occupied by biofilms formed by strains detailed in *B* on the 9-cm-diameter Petri dish was calculated and plotted. The bar represents the average of the biological repeats that are presented as individual points; the error bars are the SEM. The samples were grown at 30 °C on MSgg agar containing 50 μM FeCl_3_.

To explore how the pulcherriminic acid-minus strains expand across the substratum, we assessed if there were differences in cell organization at the outer (leading) edge of the biofilm using in situ confocal microscopy. To allow definition to be resolved in the densely packed bacterial community we mixed isogenic cells that constitutively expressed the green fluorescent protein (GFP) with nonfluorescent cells (in a 1:5 ratio). We examined the outer biofilm edge at two time points: 24 h and 120 h. No detectable phenotypic differences between wild-type and pulcherriminic acid-deficient strains were observed at 24 h of growth (Fig. 3*A*). The imaging revealed that the leading edge of the wild-type, *yvmC*, and *cypX* deletion strains contained loops of cells that extended out from the body of the biofilm biomass. The loops protruding from the biofilm were located in what appeared to be a densely packed monolayer. The cells in the monolayer looked to be composed of clonal lineages of cells as fluorescence was located in convoluted but discrete chains. In contrast, at 120 h in the wild-type biofilm, the monolayer at the leading edge of the biofilm was absent and the cells were contained within a multilayer structure that consistently presented with a tight, compact steep edge in which chains or loops of cells could not be resolved. For the *cypX* and *yvmC* deletion strains, while certain zones of the 120-h-old biofilm edge also showed steep compact edges containing multiple layers of cells (*SI Appendix*, Fig. S3), monolayers containing loops of cells that protruded from the body of the biofilm were maintained in many regions around the edge of the expanding community (Fig. 3*A*). These findings reveal a difference in the cell organization of the pulcherriminic acid-positive and -negative strains and link the presence of a monolayer of cells at the periphery of the biofilm with expansion across the surface.

Expansion of the *B. subtilis* biofilm across a surface has been shown to be driven by pressure exerted by the components in the biofilm matrix, rather than associated with flagellar-mediated motility (24). To determine whether the same processes drive radial expansion of the biofilm in the absence of pulcherriminic acid synthesis, mutations in *tasA*, *epsA-O*, *bslA*, and *hag* (which encodes flagellin) were introduced into the *yvmC* mutant (*SI Appendix*, Table S1). The footprint of the resultant biofilm was quantified after 120-h incubation and the isogenic pulcherriminic acid-positive strains were used as a reference for measuring biofilm expansion in the presence of pulcherrimin formation. We found that insertion of either the e*psA-O* or the *tasA* mutation to the *yvmC* mutant completely blocked expansion of the pulcherrimin deficient strain (Fig. 3 *B* and *C*), with the *epsA-O* and *tasA* deletion strains occupying the same limited area of the surface whether or not pulcherriminic acid was made (Fig. 3 *B* and *C*). However, for the *bslA* mutation, a small level of biofilm expansion was still attainable in the absence of BslA when pulcherrimin was not deposited into the agar (Fig. 3 *B* and *C*). Finally, the role of flagellar-based motility in expansion was tested. Consistent with previous findings (24), the footprint of the *hag* biofilm was comparable to that of the parental NCIB 3610 biofilm (Fig. 3 *B* and *C*). In contrast, while the area covered by the *yvmC hag* biofilm was significantly reduced by comparison with the *yvmC* mutant, it was greater than that occupied by the pulcherriminic acid-positive *hag* deletion (Fig. 3 *B* and *C*). The colonization the pulcherrimin nonproducing strains across a surface is therefore the consequence of a combination of flagellar-based motility coupled with expansion driven by the biofilm matrix molecules.

### Cytoplasmic Pulcherriminic Acid Does Not Trigger an Arrest in Biofilm Expansion.

We next wanted to elucidate whether cytoplasmic pulcherriminic acid or extracellular pulcherrimin was responsible for the observed biofilm growth arrest. For this purpose we needed to generate a strain where secretion of pulcherriminic acid was prevented. In *Bacillus licheniformis,* the major transmembrane transporter of pulcherriminic acid is YvmA (30) and a homolog with 59% protein identity is encoded on the genome next to *cypX* and *yvmC* in *B. subtilis*. We therefore deleted *yvmA* and assessed pulcherriminic acid secretion by monitoring pigment production. Consistent with an involvement in pulcherriminic acid secretion, the agar under cells carrying the *yvmA* deletion was a very light brown, not the rusty brown observed for the wild-type strain or the clear agar of the *cypX* and *yvmC* deletion strains (*SI Appendix*, Fig. S4). These findings are consistent with YvmA being the critical, but perhaps not sole, transporter of pulcherriminic acid to the extracellular environment. (The small amount of pulcherrimin in the agar is also consistent with limited cell lysis releasing low amounts of pulcherriminic acid into the external environment.) Having linked YvmA to release of pulcherriminic acid from the cell, we tested if the *yvmA* deletion strain formed a biofilm that was constrained in terms of the area it occupied or if the community kept expanding. The analysis determined that the biofilm formed by the *yvmA* mutant kept expanding, reaching levels similar to those measured for the *cypX* and *yvmC* strains (Fig. 2*H* and *SI Appendix*, Fig. S2*A*). These results denote that the synthesis of intracellular pulcherriminic acid in itself is not sufficient to prevent expansion of the biofilm. Rather, it is the export of pulcherriminic acid to the environment and the subsequent formation of pulcherrimin that causes biofilm growth arrest.

### Iron Limitation Occurs at the Edge of the Wild-Type Biofilm.

**Fig. 4.**
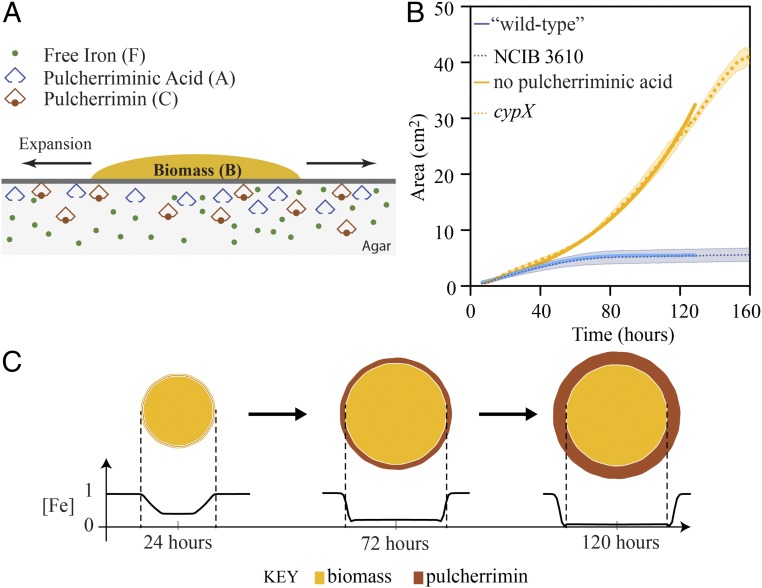
Mathematical model reveals iron limitation beyond the biofilm edge. (*A*) Schematic of the model setup. In the model the biofilm biomass *B* grows on an agar substrate containing iron and expands radially at a rate proportional to the free iron in the media *F*. Free iron is utilized for biomass production and maintenance at a rate $k_{f}BF$. Pulcherriminic acid *A* is produced and secreted by cells in the mature biofilm at a rate $k_{b}B$_._ The pulcherriminic acid chelates iron with rate $k_{p}AF$, resulting in the formation of pulcherrimin *C*. (*B*) The area of the biofilm is plotted over time. Both the biological and mathematical data are shown. The dotted line is the average of three biological repeats and shadowed area is the SEM (the NCIB 3610 and *cypX* data are from Fig. 2*H*). The solid lines show the best fit of the model in the presence and absence of pulcherriminic acid production. (*C*) Model output showing biofilm expansion and production of pulcherrimin over time (top view) and level of free iron remaining in the agar (initial level normalized to unity). Three different time points are shown.

As pulcherrimin is a complex between Fe^3+^ and pulcherriminic acid, one mechanism to explain the growth arrest of the wild-type biofilm is localized nutrient deprivation in the form of iron limitation. To test this hypothesis, we first constructed a mathematical model that incorporates the core processes of biofilm growth and pulcherriminic acid production (see Fig. 4*A* for a schematic). The biomass density B(r,t) obeys a continuity equation (conservation of mass) of the form $\partial_{t}B=-\nabla \cdot J+G$, for space ***r*** and time *t* where J=Bv is the flux with v the velocity of the biomass. *G* is the biomass production rate and encapsulates the processes of cell division, the production of extracellular matrix, and consequent water absorption. These growth processes are assumed to induce a pressure p(r,t) within the biomass that drives expansion principally in the radial direction. Hence, the velocity of the biomass at any given point is related to this internal pressure at that point via the equation v=−λ∇p, where ∇p is the gradient of the pressure and λ represents certain mechanical properties of the biofilm. This relationship is referred to as Darcy’s law in certain contexts (see, e.g., ref. 31). The biomass production rate *G* is assumed to follow a simple saturating response to iron availability. Moreover, biomass is self-generating and we assume this relationship to be linear Therefore, we write

$$G=G\left(B,F\right)≔Bg\left(F\right)\;\text{with}\;g\left(F\right)≔\frac{\kappa_{0}F^{m}}{F_{h}^{m}+F^{m}},$$

where $\kappa_{0}$ is the maximal growth rate, $F_{h}$ represents the free iron level that corresponds to half maximal growth rate, and *m* characterizes the growth response. We make the further reasonable assumption that $g\left(F\right)\equiv 0\text{for}F<F_{c}$ for some suitably chosen small value $F_{c}$ representing the minimum nutrient level capable of supporting growth processes. Substituting the expressions for ***v*** and *G* into the general conservation of mass equation yields the equation

$$\left(Biomass\;expansion\right)\partial_{t}B=\nabla \cdot \left(B\lambda \nabla p\right)+B\frac{\kappa_{0}F^{m}}{F_{h}^{m}+F^{m}}.$$ [1]

The expanding biomass interacts with its environment via (*i*) the production, export, and subsequent diffusion into the agar of pulcherriminic acid; (*ii*) the irreversible chelation of free iron by pulcherriminic acid to form pulcherrimin; and (*iii*) the utilization of free iron to support the production and maintenance of biomass. In general, the associated rate reactions can be represented respectively as

$$\left(i\right)\overset{K_{b}\left(B,F\right)}{\to }A;\;\left(ii\right)A+F\overset{K_{p}\left(A,F\right)}{\to }C;\;\left(iii\right)F\overset{K_{f}\left(B,F\right)}{\to }\emptyset ,$$

where AandCare concentrations of extracellular pulcherriminic acid and pulcherrimin, respectively, with *F* representing free iron as above. Deletion of *pchR*, a known negative regulator of *yvmC cypX* transcription (29), does not appear to increase pulcherrimin formation during biofilm formation (*SI Appendix*, Fig. S5*A*). Therefore, we conclude that pulcherriminic acid synthesis is at a maximal level in the wild-type biofilm under our conditions. Additionally, using a P*yvmC-lacZ* reporter fusion we assessed transcription from the *yvmC* promoter within the wild-type biofilm. We established that expression is essentially uniform across the biofilm with the exception of a zone of inactivity that can be observed at the periphery of younger biofilms (*SI Appendix*, Fig. S5*B*). We therefore group the processes of synthesis and transport to the extracellular space and assume a simple relationship in (*i*), namely, the rate of production of extracellular pulcherriminic acid is $K_{b}\left(B,F\right)=k_{b}B$, where $k_{b}$ represents the rate constant (which can be set to zero to mimic strains that do not synthesize pulcherriminic acid) and in (*iii*) we set $K_{f}\left(B,F\right)=k_{f}B$ with resultant utilization rate $k_{f}BF$. Finally, given the nature and distribution of the reactants *A* and *F* within an essentially homogeneous agar, we can reasonably apply the law of mass action in (*ii*) to yield a reaction rate $k_{p}FA$ for some constant $k_{p}$. Taking into account (Fickian) diffusion of the reactants in the agar (with rate constants $D_{A},D_{C},\text{and}D_{F},$respectively), we can model the above reactions using the following system of partial differential equations:

$$\begin{array}{l} \left(Pulcherriminic\;acid\right)\partial_{t}A=k_{b}B-k_{p}FA+D_{A}\Delta A,\\ \left(Pulcherrimin\right)\partial_{t}C=k_{p}FA+D_{C}\Delta C,\\ \left(Free\;iron\right)\partial_{t}F=-k_{p}FA-k_{f}BF+D_{F}\Delta F, \end{array}$$ [2]

where Δ is the diffusion (Laplace) operator.

The model is formed by coupling Eqs. **1** and **2** and solving the resultant moving boundary problem. Reflecting typical biofilm morphology, we reasonably consider expansion of the biomass to be radially symmetric with the biomass density assumed to be a constant within its footprint with the expanding outer edge defined by r≔s(t).These assumptions allow for the velocity of outer edge to be related to the pressure there via the equation

$$v=-\lambda \partial_{r}p\;\text{at}\;r=s\left(t\right).$$ [3]

The system (Eqs. **1**–**3**) can be scaled via a nondimensionalization that reduces the number of free parameters allowing for the selection of a parameter set by matching to experimental data for the mutant and wild type in turn. Model output is variously sensitive to changes to the chosen values. However, the default set is located in a region of parameter space for which the fit is good and sensitivity to change is low. Moreover, other combinations of the grouped parameters produce equally good fit to experimental data. Hence, we conclude that the modeling results presented here are not restricted to a unique selection of parameters (*SI Appendix*, Fig. S5).

With $k_{b}$ set to represent the wild-type strain, the model predicts that the radius of the biomass footprint $s\left(t\right)\propto t^{2}$, that is, the biomass area initially grows linearly (23). This linear growth phase is followed by a deceleration of biofilm expansion leading to a fixed biofilm area (Fig. 4*B*). When $k_{b}$ is reduced to zero (to model either the *yvmC* or *cypX* mutant strains), simulations again reveal a biphasic growth pattern. However, for the model mutant the initial linear growth phase is followed by exponential and seemingly unbounded expansion (Fig. 4*B*).

The model predicts that arrest results directly from the chelation of iron. For the wild type, the production of A(r,t) within the footprint of the biomass coupled to its subsequent diffusion into the surrounding region results in $F\left(r,t\right)<F_{c}$ within a region $0\leq r<r_{h}\left(t\right)$ with $s\left(t\right)<r_{h}\left(t\right)$ where the inequality is strict and $F_{c}$ is the growth threshold defined above. Consequently, $g=0\;\text{for}\;0\leq r<r_{h}\left(t\right)$ and hence from from Eq. **3** and after a little calculation, v=0 at r=s(t). Finally, the model wild-type scenario determines that $C\left(r,t\right)>C_{c}\;\text{for}\;0\leq r<r_{h}\left(t\right).$ On the contrary, for the mutant $\left(k_{b}=0\right)$ local utilization of iron for growth is not predicted to be sufficient to reduce F(r,t) to subcritical levels in a region $s\left(t\right)\leq r<r_{h}\left(t\right)$. Hence g>0 and therefore v>0 at >r=s(t).[In this case, C(r,t)≡0, as would be expected.]

Therefore, the minimal mathematical model (Eqs. **1**–**3**) is capable of capturing the global growth characteristics that were measured experimentally for both the wild-type strain and those unable to synthesize pulcherriminic acid. The output of the model qualitatively supports our hypothesis that arrest of biofilm expansion is mediated by a simple process that is influenced by just one molecule, pulcherriminic acid. The model predicts that as the pulcherriminic acid diffuses, it forms a radially expanding wave that overtakes the leading edge of the biofilm, culminating in a zone of iron limitation in the agar just beyond the expanding biofilm edge leading directly to the arrest of biofilm expansion. Moreover, it predicts that pulcherrimin should be visible within the growth medium not only under the footprint of the biomass but also within a “halo” that extends beyond the leading edge and links the appearance of this phenomena directly to expansion arrest (Fig. 4*C*).

**Fig. 5.**
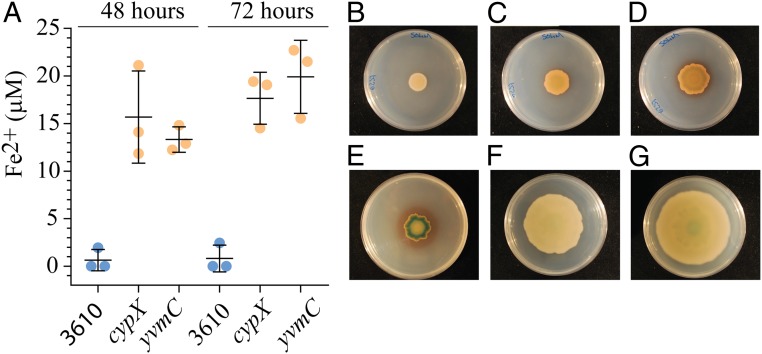
Detection of iron depletion is heterogeneous in the population. (*A*) The level of available ferrous and ferric iron in the agar under the colony was detected using a ferrozine assay. The bar represents the average of the biological repeats that are presented as individual points; the error bars are the SD. Biofilms formed by NCIB 3610 and the *cypX* (NRS5532) and *yvmC* (NRS5533) deletion strains were grown at 30 °C on MSgg agar containing 50 μM FeCl_3_ for the times indicated before analysis of the agar. (*B*–*G*) Images of strains containing the P*dbh*A-*lacZ* transcriptional reporter fusion after growth at 30 °C on MSgg agar in 9-cm-diameter Petri dishes containing 120 μg⋅mL^−1^ X-Gal. (*B–E*) The 3610 P*dbh*A-*lacZ* after 24, 48, 72, and 120 h of incubation, respectively. (*F*) *cypX* P*dbh*A-*lacZ* at 120 h of incubation. (*G*) *yvmC* P*dbh*A-*lacZ* at 120 h of incubation. The image shown is representative of three biological repeats.

To test the “depletion wave” hypothesis, we measured the level of available iron in the agar where the pulcherrimin was located after 48 and 72 h (Fig. 5*A*). While the uncolonized agar retained an iron level of ∼50 μM, the level of available iron in the agar where the pulcherrimin was deposited dropped to below the detection limit of the assay (<1 μM) by 48 h (Fig. 5*A*). In contrast, in an equivalent area of agar taken from under the biofilm formed by the *yvmC* and *cypX* deletion strains, ∼15 to 20 μM iron remained, even after 72-h incubation (Fig. 5*A*). To test whether the cells responded in distinct ways to these different iron levels we used a P*dhbA-lacZ* transcriptional reporter fusion. The *dhbA* gene encodes a protein needed for the biosynthesis of the siderophore bacillibactin which is synthesized under conditions of iron limitation (32, 33) (*SI Appendix*, Fig. S6). For the wild-type strain evidence of activity from the P*dhbA-lacZ* transcriptional reporter could not be detected at early time points of biofilm formation (up to 48 h) (Fig. 5 *B* and *C*). However, around 72 h, the time at which the cells cease expanding across the surface, a ring of cells with a deep blue color developed near the periphery of the biofilm, which is indicative of *dhbA* transcription (Fig. 5*D*). This pattern of coloration was sustained and enhanced in the biofilm at later time points (Fig. 5*E*). In contrast, for both strains that do not form pulcherrimin, very little or no blue color was visible in the biofilm even after 120 h, indicating that transcription of the P*dhbA-lacZ* fusion was negligible (Fig. 5 *F* and *G*). Thus, the wild-type biofilm experiences the iron deprivation particularly toward the edge of the biomass, whereas the pulcherrimin-deficient strains are not exposed to the same degree of iron limitation. Collectively these findings show that while cell growth consumes iron from the agar, it is pulcherrimin production that results in the reduction of free iron in the biofilm vicinity to levels that cannot support continued expansion.

### Expansion of the Biofilm Is Restricted at Extremes of Iron Levels.

Based on the data presented above we explored whether increasing the level of iron in the growth medium could overcome the arrest in biofilm expansion seen for the wild-type strain. We first tested this using the mathematical model by simply scaling the initial free iron concentration $F\left(r,t=0\right)\equiv F_{0}$. The output revealed that increasing $F_{0}$ modulated the biphasic growth profile, leading to a longer initial linear phase (*SI Appendix*, Fig. S7*A*) with the terminal area occupied by the biofilm an increasing function of the initial iron level (*SI Appendix*, Fig. S7*B*). Moreover, the model predicted that increasing $F_{0}$ also constrains the pulcherrimin halo closer to the footprint of the biomass both during expansion phase and at the terminal growth point (*SI Appendix*, Fig. S7*C*). We note that at first glance this seems somewhat counterintuitive. However, it is in fact due to the chelation rate $-k_{p}AF$ in the first equation of [**2**] dominating production and diffusion of A in the halo zone.

**Fig. 6.**
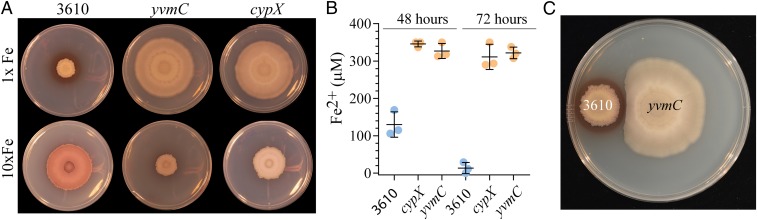
Pulcherriminic acid production modulates local iron levels. (*A*) NCIB 3610 and the *cypX* (NRS5532) and *yvmC* (NRS5533) deletion strains were grown at 30 °C on MSgg agar containing either 50 μM FeCl_3_ (1×) or 500 μM FeCl_3_ (10×) for 120 h before imaging. (*B*) The level of available ferrous and ferric iron in the agar was detected using a ferrozine assay. The bar represents the average of the biological repeats that are presented as individual points; the error bars are the SD. The starting concentration in the agar was 500 μM FeCl_3_ and the maximum detection limit in these conditions was 350 μM. (*C*) Biofilms of NCIB 3610 and *yvmC* (NRS5533) were inoculated onto a 15-cm-diameter MSgg (1.5% wt/vol agar) plate and incubated at 30 °C for 120 h before imaging.

Following the trend of the mathematical predictions, our experimental data show that when the level of FeCl_3_ in the growth medium was raised from 50 μM to 500 μM the footprint of the biofilm formed by NCIB 3610 also increased (Fig. 6*A* and *SI Appendix*, Fig. S7*D*). Moreover, in broad agreement with the mathematical prediction, the pulcherrimin halo was reduced. In fact no distinct halo of pulcherrimin protruded beyond the edge of the biofilm. We also noted that pulcherrimin was prevalent both within the biomass of the biofilm (which was dark pink in color) and in the agar. The depletion of iron from the growth medium upon production of pulcherrimin was substantial. When we measured the level of available iron in the agar underneath the wild-type biofilm grown on a starting FeCl_3_ concentration of 500 μM, it was ∼120 μM at 48 h and dropped to below the detection limit of the assay by 72 h (Fig. 6*B*). These data demonstrate that increasing the iron in the environment can overcome the impact of iron chelation by pulcherriminic acid.

In contrast to the rapid depletion of iron in the agar in the vicinity of the wild-type strain, we found that the agar under the biomass of *yvmC* and *cypX* deletion strains retained ∼300 to 350 μM iron, even after 72-h incubation (Fig. 6*B*). Additionally, and again contrary to that of the wild-type strain, analysis of biofilm formation by the *yvmC* and *cypX* deletion strains revealed that the 10-fold increase in the FeCl_3_ level resulted in a reduction of the footprint area occupied after 120 h (Fig. 6*A* and *SI Appendix*, Fig. S7*D*). Based on these findings, we hypothesized that the negative impact on footprint area of increasing iron concentration in the medium could be a consequence of the level of iron in the growth medium being toxic for the *yvmC* and *cypX* deletion strains. However, analysis of cells grown in planktonic conditions revealed that the growth rates of the *yvmC* mutant was broadly similar to that of the wild type (*SI Appendix*, Fig. S7*E*). Thus, the lack of biofilm expansion in the presence of 500 μM FeCl_3_ for strains that do not produce pulcherriminic acid is not a consequence of toxicity. Therefore, we conclude that when the level of iron is above a threshold the cells are prevented from triggering expansion of the biofilm. When these findings are coupled with the self-restriction of growth of the wild-type strain when cultured on 50 μM FeCl_3,_ they indicate that if the level of available iron in the local environment is either too low or too high expansion of the biofilm is restricted.

### Protection of a Niche through Pulcherrimin Formation.

As pulcherrimin restricts growth through local depletion of iron, we hypothesized that production of pulcherriminic acid might be advantageous to protect a local niche. We therefore explored if the pulcherrimin deposit produced by the wild-type biofilm could restrict the growth of neighboring populations. When the wild-type and *yvmC* mutant strain were inoculated onto the same plate, we found that the otherwise unlimited growth of the *yvmC* biofilm was restricted just in the proximity of the pulcherrimin in the agar (Fig. 6*C*). In the analogous experiment where the *yvmC* mutant was grown adjacent to another biofilm formed by the *yvmC* mutant there was no zone of growth inhibition (*SI Appendix*, Fig. S7*F*), with cells growing abutted to each other. Thus, pulcherrimin-forming populations can protect their niche from colonization by neighboring bacteria.

### Comparative Genome Analysis.

**Fig. 7.**
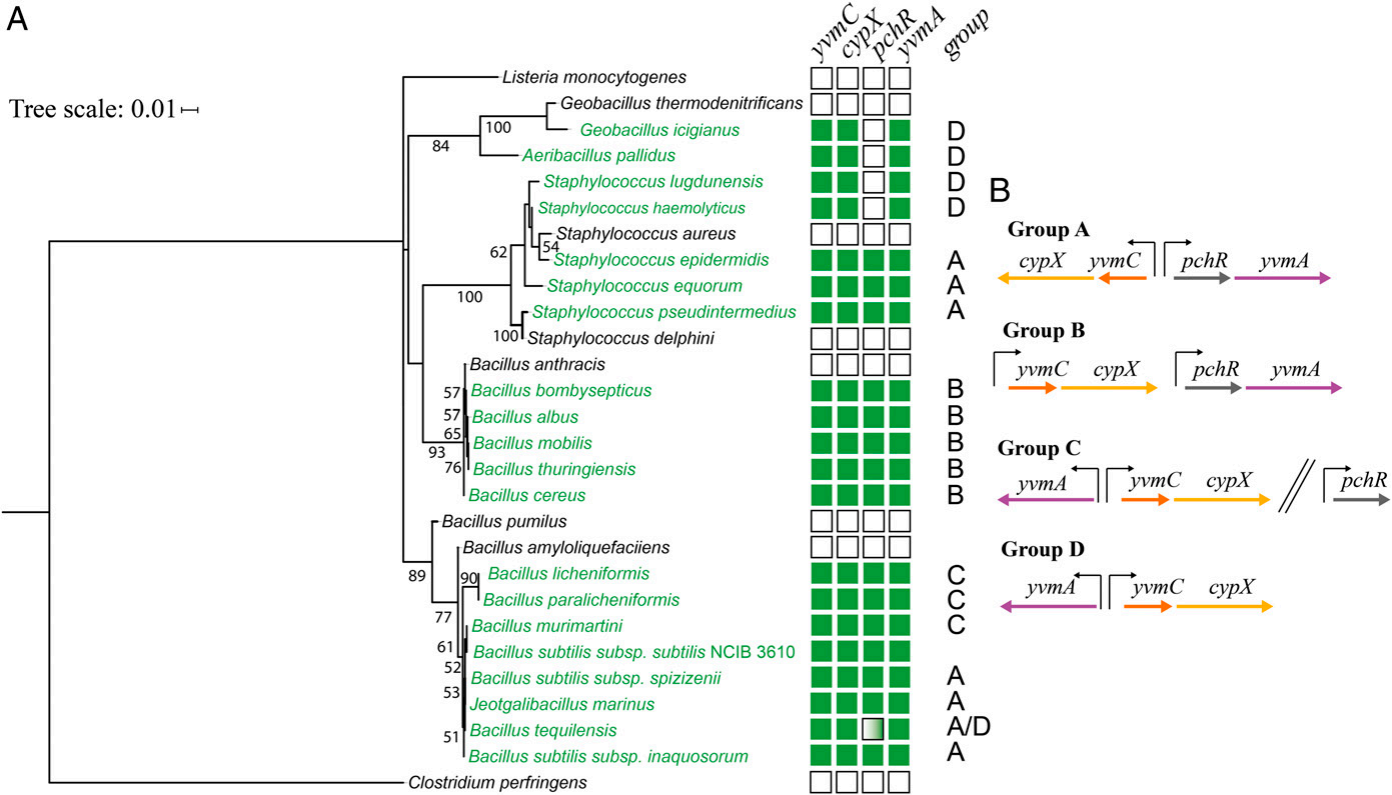
Comparative genomic analysis of the pulcherriminic acid biosynthetic cluster. (*A*) Phylogenetic tree showing species that contain genes within the pulcherriminic acid biosynthetic cluster. The numbers adjacent to branches indicate the bootstrap values for 350 replicates. Species with members that contain the cluster are shown in green and those without the genes in black. The boxes on the right-hand side show the genes that were identified in the genomes; green is positive and white is negative. The dual green/white box indicates variability in the presence of the gene throughout the genomes. The box containing the letters A–D indicates the genomic organization of the genes in the cluster. (*B*) The organization of the pulcherriminic acid biosynthetic genes as identified through comparative genome analysis. The black upright arrows represent promoters and the two parallel slanted lines indicate a gap in the genome between the gene.

Pulcherrimin has been observed to be made by both eukaryotic and prokaryotic microorganisms (27, 34). It is, however, only more latterly that the biosynthetic pathways involved have been elucidated (35, 36). We conducted comparative genomic analysis that revealed the genes needed for pulcherriminic acid synthesis and secretion (*yvmC*, *cypX*, and *yvmA*) were found in the genome or on plasmids of species beyond *B. subtilis*. It should be noted that the pulcherrimin biosynthetic cluster is not found in all isolates of each species (including *B. subtilis*), but those bacteria that contain the genes include isolates of *Bacillus cereus*, *Bacillus thuringenesis*, and *Staphylococcus epidermidis* species (Fig. 7 *A* and *B*). Differences in gene synteny from that of the *B. subtilis* genome were apparent with the regulator, *pchR*, not always being present in the genome and additionally the organization of the remaining genes was varied (Fig. 7*B*). However, one consistent feature in the gene organization pattern was that the *yvmC* coding region always preceded that of *cypX*. The patchy distribution of the genes both within a single species and between closely related bacteria suggests loss of the genes over time, rather than reoccurring acquisition. This pattern of gene loss and distribution is consistent with the profile observed in yeast (35). However, given the knowledge that at least two routes have evolved for pulcherriminic acid production (35, 36), we cannot exclude the possibility that other species also synthesize pulcherriminic acid using an as-yet-unidentified pathway.

## Discussion

By coupling mathematical modeling and microscopy with bacterial genetics, we have revealed that growth arrest is a distinct stage of *B. subtilis* biofilm development that is not simply a consequence of spore formation. Our conclusion that cells in the biofilm retain metabolic activity is consistent with work demonstrating that residents of the *B. subtilis* biofilm undergo adaptation in mature biofilms which results in changes to the genome (37). We identify that production of pulcherriminic acid underpins growth arrest of the biofilm. However, its synthesis is not sufficient; it is the chelation of Fe^3+^ through the formation of pulcherrimin surrounding the population on the biofilm that is needed to self-restrict biofilm expansion. Self-restriction of growth is counterintuitive from an evolutionary perspective and this led us to explore possible advantages associated with formation of pulcherrimin. Our analyses revealed beneficial effects for the pulcherrimin-producing bacterium as the pulcherrimin extending outside the biomass of the biofilm can prevent colonization by neighboring communities. We also uncovered that pulcherrimin formation confers an advantage through modulation of the external conditions in high-iron environments. Together these data shine light on the seeming paradox of pulcherrimin-mediated self-restriction of expansion.

### Growth in the Biofilm Is Controlled by Iron Levels.

Iron is a micronutrient that is essential to support bacterial growth and survival, but while it is relatively abundant in the soil it is largely inaccessible (38). The low accessibility of iron within the soil stimulates competition between plants and microbes for its acquisition. Bacteria have evolved many mechanisms to capture and sequester iron from the environment including iron uptake systems (39), siderophore production (32), and sorption methods (40). Here we have uncovered the contribution that iron chelation, mediated by pulcherrimin formation, has on the physiology of the *B. subtilis* biofilm. Indeed, we have shown that chelation is the dominant cause of free iron depletion from the medium—not utilization for growth itself. At low iron levels pulcherriminic acid diffuses beyond the expanding biofilm leading edge, chelating Fe^3+^ and self-restricting expansion of the biomass. These findings are consistent with data linking *B. subtilis* biofilm formation to iron availability (41, 42). Consistent with iron levels’ being the dominant factor influencing biofilm expansion, both mathematical and experimental data show that increasing the iron concentration in the growth medium overrides this growth inhibition. When iron is plentiful, the profile of pulcherrimin deposition alters and a halo surrounding the cells is not apparent. This means that the leading edge of the biofilm is not exposed to iron limitation and continues to expand for longer. The molecular mechanisms controlling the transition of *B. subtilis* to a nonexpanding phase when iron levels are too low need further elucidation. Moreover, whether there is a feedback loop between the concentration of iron in the environment and pulcherriminic acid production, or if pulcherriminic acid biosynthesis is independent of the level of iron when in a biofilm, needs further investigation. There is precedent for a feedback loop where pulcherriminic acid levels are influenced by environmental iron concentrations: in *B. licheniformis* transcription of two genes linked with controlling transcription from the *yvmC* promoter are responsive to iron (29, 30). The possibility of posttranscriptional regulation of pulcherriminic acid production by the level of iron in the environment is additionally supported by the identification of an extended 5′ untranslated region upstream of the *yvmC* coding region (43). Such regions are typical of riboswitches that can bind a wide array of metabolites and metals and control gene transcription and translation efficiency for example (44, 45).

### Sequestration of Iron in Biofilms.

The ability to modulate iron levels in the context of biofilm formation appears to be a theme emerging in the literature. While in this study we have uncovered the involvement of iron chelation by pulcherrimic acid in growth arrest, the accumulation of ferric and ferrous ions in the matrix of *B. subtilis* has been linked with protection from erosion and toxicity (46). The sequestration of iron within the extracellular matrix is not unique to *B. subtilis*, as in biofilms formed by the gram-negative bacterium *Pseudomonas aeruginosa* the Psl polysaccharide fibers of the matrix bind and sequester both ferric and ferrous ions. This is not a passive process, as high levels of iron in the environment trigger Psl synthesis and therefore promote accumulation of iron in the local environment of the bacterial population (40). Similarly a polysaccharide produced by *Klebsiella oxytoca* biofilms has also been shown to bind iron (47) and electron-dense iron-enriched deposits accumulate in the fibrous matrix of *Enterococcus faecalis* biofilms (48). In *E. faecalis* the deposits promote extracellular electron transport by acting as an electron sink and thus support growth in the biofilm through altered metabolism. Therefore, the purpose of controlling iron levels in the microenvironment of the biofilm is varied and is likely to be specific for different species.

### Pulcherriminic Acid-Mediated Chelation of Iron.

In yeast strains, such as *Metschnikowia pulcherrima*, pulcherrimin formation has been linked with antagonism against other species (49). In *B. subtilis* the function of pulcherrimin was entirely unknown. We have identified that pulcherriminic acid allows *B. subtilis* to alter the level of freely available iron in its vicinity, over (at least) two orders of magnitude, as an initial concentration of 500 μM FeCl_3_ in the growth medium can be entirely sequestered within 72 h (Fig. 6*B*). This remarkable ability to manipulate the external environmental conditions has corresponding benefits. For example, the pulcherrimin halo of the wild-type strain prevents invasion of its niche by bacteria found in the surrounding environment (Fig. 6*C*). This niche protection mechanism is likely to be effective against any microorganism that depends on iron for growth. Consistent with this, pulcherrimin production has been associated with biocontrol processes (50). However, this long-held interpretation of how pulcherrimin functions has been challenged by the recent classification of pulcherriminic acid as a siderophore in the yeast *Kluyveromyces lactis* (35). In *K. lactis* a membrane bound protein encoded by the gene PUL3 is linked with utilization of pulcherrimin via uptake. This opens up the possibility that pulcherrimin producers across both bacterial and eukaryotic microbes are exploited by neighboring “cheater” cells. In this context, the nonproducing cells could make use of the chelated iron but would not contribute to its deposition (35). In nature, the routes used for iron acquisition are highly diverse and competition between ecologically distant species is common. For instance, insect herbivores are able to hijack plant iron acquisition systems (51) and the soil bacterium *Streptomyces venezuelae* produces a volatile compound (trimethylamine) that enhances colonization by the bacterium through modulating iron availability (52, 53). Therefore, while there is no evidence that *B. subtilis* is able to use pulcherriminic acid as a siderophore, the intriguing question of how pulcherrimin is accessed and utilized in the complex soil environment remains open to multiple possibilities.

## Materials and Methods

### General Growth Conditions and Strain Construction.

The *B. subtilis* strains used and constructed in this study are detailed in *SI Appendix*, Table S1. *B. subtilis* 168 derivatives were obtained by transformation of competent cells with plasmids using standard protocols (54). SPP1 phage transductions were used to introduce DNA into *B. subtilis* strains (55). *Escherichia coli* strains were routinely grown in lysogeny broth (LB) medium (10 g NaCl, 5 g yeast extract, and 10 g tryptone per L) at 37 °C for 16 h. When appropriate, antibiotics were used at the following concentrations: ampicillin 100 μg⋅mL^−1^, erythromycin 1 μg⋅mL^−1^, kanamycin 25 μg⋅mL^−1^, neomycin 8 μg⋅mL^−1^, and spectinomycin 100 μg⋅mL^−1^.

### Plasmid Construction.

Details of plasmid construction methods are provides in *SI Appendix*, *Supplementary Information Text*.

### Biofilm Formation.

*B. subtilis* strains were grown on MSgg medium (5 mM potassium phosphate and 100 mM Mops at pH 7.0 supplemented with 2 mM MgCl_2_, 700 μM CaCl_2_, 50 μM MnCl_2_, 1 μM ZnCl_2_, 2 μM thiamine, 0.5% glycerol, and 0.5% glutamate) (9) solidified with 1.5% select agar (Invitrogen) at 30 °C at the indicated time points. FeCl_3_ was added at the indicated concentration and where not specified was used at 50 μM. When appropriate 5-bromo-4-chloro-3-indolyl β-d-galactopyranoside (X-Gal) was added at 120 μg⋅mL^−1^. To set up a biofilm, a 3-mL aliquot of LB medium was inoculated with an individual colony taken from an overnight plate and grown at 37 °C to an OD_600_ of 1. Unless otherwise stated, 5 µL of the culture was placed onto an MSgg plate which was incubated at 30 °C for morphology and hydrophobicity studies. Images of colony biofilms were recorded using a Nikon D3200 digital camera mounted on a Kaiser RS3XA copy stand or using a Leica MZ16FA stereomicroscope.

### Footprint Area Measurements.

Images were taken using either a five-megapixel USB autofocus camera module CAM8200-U controlled with Python-Bash software developed specifically or using a BioRad GelDoc XR+. Images were analyzed with Fiji (56) using standard thresholding methods (57) to extract the biofilm footprint area occupied.

### Sporulation Assay.

For heat-resistant spore quantification, colony biofilms were grown for 24, 48, 72, 96, or 120 h at 30 °C. The complete biofilm was harvested and suspended in 1 mL of saline solution. The biomass was disrupted by passage through a 23 × 1 needle eight times and subsequently subjected to mild sonication (20% amplitude, 1 s on, 1 s off for 5 s total) to liberate bacterial cells from the matrix. To kill vegetative cells, the samples were incubated for 20 min at 80 °C. To determine the percentage of spores, serial dilutions were plated before and after the 80 °C incubation on LB agar supplemented with 25 μg⋅mL^−1^ kanamycin. The percentage of spores was established by colony forming unit counting and results are presented as the percentage of colony forming units obtained after incubation of the samples for 20 min at 80 °C divided by the number of colony forming units obtained before the heat inactivation.

### Biofilm Hydrophobicity Imaging.

Biofilm hydrophobicity was determined by placing a 5-µL droplet of water on the upper surface of biofilms that had been grown for 48 h at 30 °C (17). The water droplet was allowed to equilibrate for 5 min before imaging using a ThetaLite TL100 optical tensiometer (Biolin Scientific).

### Ferrozine Assay.

The level of Fe^2+^ present in the agar was measured as follows. A “punch” of agar was taken using the reverse side of a 200-μL pipette tip and the extracted agar was expelled into a well of a 96-well plate. The absorbance at 562 nm was measured and recorded to provide the baseline. The assay was started by addition of 63 μL of the master mix that comprised 46.7 μL of 1 M potassium acetate (pH 5.5 with acetic acid) and 3.3 μL 1 M ascorbic acid to reduce available Fe^3+^ to Fe^2+^ and 13 μL ferrozine (stock 50 mg/mL in 1 M potassium acetate). The samples were incubated at 37 °C for 1 h before reading absorbance at 562 nm using a plate reader. A standard curve for the assay was generated using agar punches extracted from MSgg agar plates prepared with defined concentrations of FeCl_3_ that ranged from 0 to 400 μM. The maximum value of Fe^2+^ that could be measured using this method was 350 μM, and therefore samples that presented as above this value were given a value of 350 μM as this represents the upper limit of the assay. The data presented are an average of three technical samples for each condition.

### Confocal Microscopy.

Four milliliters of MSgg medium supplemented with 1.5% (wt/vol) agar was placed into a 35-mm-diameter Petri dish and dried for 1 h in a laminar flow hood. NCIB 3610, ∆*cypX*, and ∆*yvmC* strains and their respective constitutive GFP-producing daughter strains (*SI Appendix*, Table S1) were grown separately in LB medium to an OD_600_ of 1. The cultures were then mixed to produce a suspension of cells where 80% were nonfluorescent and 20% GFP-expressing. One microliter of this mixed culture solution was spotted into the center of the Petri dish and was incubated at 30 °C for the indicated time period.

A Leica SP8 upright confocal was used to image the edge of the biofilm using a 10× 0.3 N.A. air objective and a heated chamber that was prewarmed to 30 °C. A cling film tent was draped from around the objective and tucked loosely under the stage to eliminate airflow across the plate and minimize dehydration (and therefore shrinkage) of the agar. An additional 35-mm-diameter Petri dish was filled with water and placed next to the biofilm plate to increase the humidity inside the tent. An argon-ion laser was used to excite the GFP at 488 nm and 2% power. Z-stacks capturing the full height of the biofilm border were specified based on the presence of GFP-containing cells and planes of 1,024 × 1,024 pixels were acquired quickly using a resonant mirror, averaging 16 scans per line. Data were imported into an OMERO (58) server and figures were prepared using OMERO.figure (http://figure.openmicroscopy.org/). The raw data are available (59).

### Bioinformatics Analysis.

Genome sequences of 44,046 strains, representing 8,244 species, were downloaded from Ensembl Bacteria release 40 (60) and supplemented with the sequence of *Staphyloccocus delphini* 8086 (PRJEA8701; ENA Release 127). Nucleotide sequences of the genes comprising the pulcherriminic acid biosynthetic cluster were obtained from *B. subtilis* BS49 (GCA_000953615.1; ENA Release 127) and corresponded to the following coordinates on the genome: *cypX* (negative strand) 3638633–3639850; *yvmA* (positive strand) 3641568–3642779; *yvmB* (also known as *pchR*) (positive strand) 3641038–3641547; and *yvmC* (negative strand) 3639866–3640612. The nucleotide sequenced were translated to peptide sequences using the transeq program from the EMBOSS package version 6.5.7.0 (61). A similarity search of the database was carried out for each gene using the tblastn program of NCBI BLAST+ 2.5.0 (62) using an e-value cutoff of 0.05. The resulting alignments were parsed using a custom biopython (63) script to identify strains with sequences where >50% of the length of the query sequence was represented. The orientation and organization of the genes within the cluster were also compared in isolates carrying sequences with similarity to all four members of the cluster to identify isolates carrying the sequences on the same contig sequence, and the relative orientation of these. Our analysis revealed that the *pchR* gene, which encodes a transcription regulator, was not always found in the same genomic location (group C) as the other genes in the biosynthetic cluster and in some cases was missing from the genome (group D) (Fig. 7*B*). Therefore, we deemed *yvmC, cypX*, and *yvmA* as the core cluster and included strains where *pchR* was absent in our analysis (Fig. 7*A*). The source of each sequence (chromosomal or plasmid DNA) was determined from annotations in the database entries, where appropriate annotations were provided. To resolve nonspecific taxonomic assignments in the databases, 16S rRNA gene sequences from all species were identified in the genome sequences by BLASTN searches using the *B. subtilis* BS49 16S rRNA as a query and the resulting sequences classified using ExBioCloud.net (64) (May 2017 database release) (*SI Appendix*, Table S2). To construct the phylogenetic tree the 16S rRNA sequences were aligned by MAFFT v7.407 (65) using the L-INS-i algorithm. A maximum likelihood tree was generated using RAxML 8.2.12 (66) using the GTRGAMMA model and rapid bootstrapping analysis with automatic frequency-based criteria. *Clostridium perfringens* was included as an out group in the analysis and used to root the tree.

### Mathematical Model.

Full details of the mathematical model are provided in *SI Appendix*, *Supplementary Information Text* and Table S3.

## Supplementary Material

Supplementary File
