# Supplementary material for: Pulcherrimin formation controls growth arrest of the Bacillus subtilis biofilm
Source: Proc Natl Acad Sci U S A. 2019 Jun 19;116(27):13553–62. doi: 10.1073/pnas.1903982116 (PMC6613138; doi:10.1073/pnas.1903982116)
Supplement: Supplementary File [file pnas.1903982116.sapp.pdf]

PNAS
www.pnas.org

Supplementary Information for

Pulcherrimin formation controls growth arrest of the *Bacillus subtilis* biofilm

Sofia Arnaouteli, Daniel Matoz-Fernandez, Michael Porter, Margarita Kalamara, James Abbott, Cait E. MacPhee, Fordyce A. Davidson, Nicola R. Stanley-Wall

To whom correspondence should be addressed:
Prof. Nicola R. Stanley-Wall
Email: n.r.stanleywall@dundee.ac.uk
or
Prof. Fordyce A. Davidson
Email: f.a.davidson@dundee.ac.uk

This PDF file includes:

Supplementary text
Figs. S1 to S7
Tables S1 to S3
References for SI reference citations

www.pnas.org/cgi/doi/10.1073/pnas.1903982116

Supplementary Information Text

Full details of the mathematical model

Model Scaling: Spatial and temporal scales ensure that it is reasonable to assume that the biofilm can be modelled as planar and within the expanding outer edge, $B \equiv B_s$ for some constant B_s. Beyond the edge of the biomass, $B \equiv 0$ and hence the growth pressure $p \equiv 0$. Scaling also ensures that it is reasonable to assume there are no gradients in the z −direction. Finally, growth experiments demonstrate a high degree of radial symmetry. Therefore the model can be reduce further to a 1-dimensional spatial domain $0 \leq r \leq R$ where the space variable r represents radial distance from the centre of the expanding biomass and R represents the radius of the Petri dish. Recall that $r = s(t)$ marks the leading edge of the advancing biomass and we consider $0 < s(t) < R$. Systems [1] and [2] become

$$
\begin{aligned}
&0 = \frac{1}{r}\partial_r(\lambda\, r\, \partial_r p) + g(F),\\
&\partial_t A = k_b B_s - k_p\, A\, F\; + D_A\, \frac{1}{r}\, \partial_r\, (r\, \partial_r A),\\
&\partial_t C = k_p\, A\, F\; + D_C\, \frac{1}{r}\, \partial_r\, (r\, \partial_r C),\\
&\partial_t F = -k_p\, A\, F - \; k_f F\, B_s\; + D_F\, \frac{1}{r}\, \partial_r\, (r\, \partial_r F) \qquad \text{for} \in (0, s)\;, t > 0 \qquad [5]
\end{aligned}
$$

and

$$
\begin{aligned}
&0 = p,\\
&\partial_t A = -k_p\, A\, F\; + D_A\, \frac{1}{r}\, \partial_r\, (r\, \partial_r A)\\
&\partial_t C = k_p\, A\, F\; + D_C\, \frac{1}{r}\, \partial_r\, (r\, \partial_r C),\\
&\partial_t F = -k_p\, A\, F\; + D_F\, \frac{1}{r}\, \partial_r\, (r\, \partial_r F), \qquad \text{for} \in [s, R)\;, t > 0\;. \qquad [6]
\end{aligned}
$$

Appropriate conditions at the leading edge (moving boundary) are

$p = 0;\ \partial_r C = 0;\ \partial_r A = 0$ and $\partial_r F = 0$ at $r = s(t)$. [6]

The moving boundary itself is defined by equation [4]. Initial data corresponding to a localised inoculum are:

$F \equiv F_0, \qquad C = A \equiv 0, \qquad r \in [0, R];$

$p = p_0(r), \qquad r \in [0, s(t_0));$

$p = 0, \qquad r \in [s(t_0), R].$

The following non-dimensionalisation can be performed

$$\hat{t} = \frac{t}{T}, \hat{r} = \frac{r}{L}, \hat{B} = \frac{B}{B_s}, \hat{A} = \frac{A}{A_s}, \hat{F} = \frac{F}{F_s}, \hat{C} = \frac{C}{P_s}\;, \hat{p} = \frac{p}{p_s}, \hat{v} = \frac{v}{v_s}.$$

Setting $B \equiv B_s,\ p_s = \frac{\kappa_0 L^2}{\lambda}\,\hat{\lambda},\ v_s = L\kappa_0,\ A_s = F_s = C_s = F_h$ where F_h is the level of free iron that results in the growth rate being half its maximal value results in a new set of grouped (non-dimensionalised) parameters given by

$$\hat{B} \equiv 1, \quad \widehat{k_b} = \frac{Tk_b}{F_h}\,B_s,\ \widehat{k_p} = k_p T F_h,\ \widehat{k_f} = k_f T\,B_s,\ \hat{\lambda} = \frac{\lambda}{\kappa_0 L^2}\,p_s.$$

Our experiments suggested an appropriate time and length scale to be T = 250 mins and L = 1 mm capturing the observed initial maximal growth rate. Moreover, a reasonable estimation for the diffusion of pulcherriminic acid within media considered here is $D_A \sim 10^{-2}\text{mm}^2\text{min}^{-1}$. We therefore set $\kappa_0 = \frac{1}{T} = \frac{1}{200}$ mins^{-1} and $\widehat{D}_A = 10$. We make the reasonable assumption that the diffusion of free iron and pulcherrimin in the agar to be orders of magnitude smaller than that for pulcherriminic acid (this was subsequently confirmed by observing that a region of depleted iron is maintained within the footprint of the biofilm long after expansion has stopped). In the non-dimensional setting, equation [5] becomes

$$0 = \frac{1}{r}\partial_r(\lambda\, r\, \partial_r p) + \frac{F^m}{1 + F^m},$$

where we have dropped the hats for ease of exposition. Table S4 summarises the variables and parameters and the non-dimensional value used in the simulations (again dropping hats).

Model Solution

The non-dimensional system was solved using a finite difference method in space and time in an iterative manner to account for the moving boundary. At each step, the edge of the biomass was extended by a small amount governed by its velocity as computed by Eqn. [5]. The well-known MATLAB solver pdepe (see e.g. https://uk.mathworks.com) was iteratively employed to implement a finite difference method of lines approach to produce solutions to each new (fixed) boundary value problem.

Scheme:

1. Set $t = t_0$ and set $s(t) = 1$. Choose a small number $0 < \delta \ll 1$.
2. Prescribe the initial data with $p = p_0(r) > 0$ and $F \equiv F_0 > 0$ constant.
3. Compute $v = -\lambda\, \partial_r p$ at $r = s(t)$.
4. Set $s(t + \delta\, t\,) = s(t) + v\delta t$.
5. Solve for $p,\ C,\ A,\ F$ with $(r, t) \in [0, R] \times (0, t + \delta\, t]$.
6. Set $t = t + \delta\, t$ and return to 3.

Model Parameters

A best fit parameter set was formed by matching to experimental data as follows (Table S3). Parameters k_f, m and λ were fitted to experimental data. With these now set, k_b and k_p were fitted. All system parameters are positive constants, which take a single value for all the simulations ($k_b$apart). For computational purposes, we assume the extracellular pulcherriminic acid production rate k_b to a constant – either some positive value (wild-type) or zero (mutant) within the support of the *mature* biomass (i.e. within the moving boundary) and zero outside. Here, mature biomass is defined as biomass at time *t* whose radius lies within the interval $[0, s(t-\delta t)$ where δt is defined in the scheme above. This model the existence of a peripheral region of biomass just behind the leading edge which does not produce and/or export pulcherriminic acid. The presence of this peripheral zone is necessary to obtain expansion arrest in the model and moreover is directly in line with experimental observations of such a zone in the wild type (Fig S5).

A parameter sensitivity analysis was conducted (see Fig S5C). The relative error

$$E := \frac{1}{N}\sum_{i=1}^{N}\frac{\left|area_i^{exp} - area_i^{model}\right|}{area_i^{exp}}$$

was computed by solving the system of equations fitting to the area data for the pulcherrimin mutant and the wild-type. 170 combinations of m and k_f were fitted to mutant data and 90 values of k_b and k_p were fitted to the wild type. 16 values of D_f and 14 values of the D_a were fitted to the mutant and wild-type in turn. These plots reveal regions of low error and low sensitivity: dark blue regions (low error) cover sizable proportions of parameter space (low sensitivity) in the vicinity to the chosen value.

Materials

Plasmid Construction.

Genetic complementation of Δ*yvmC* and Δ*cypX* was achieved by PCR amplification of the *yvmC*-*cypX* region, including 500 base pairs upstream of the *yvmC* coding region (using primers NSW2461 (5'- ATGC<u>GAATTC</u>TCATTAAGGTGCAGCAGTCTC-3') and NSW2456 (5'- ATG<u>CGCATG</u>CTTATGCCCCGTCAAACGCAA-3')), from NCIB 3610. Fragment was digested using the EcoRI/SphI restriction enzymes and the purified fragment was ligated into pDR111. The resulting plasmid pNW1722 was introduced to *B. subtilis* strain 168 at the *amyE* locus by virtue of the spectinomycin resistance cassette. The

complementation construct was subsequently transferred to the NCIB 3610 derived strains Δ*yvmC* (NRS5533) and Δ*cypX* (NRS5532) using SPP1 phage transduction.

Activity from the *yvmC* promoter was monitored using production of β-galactosidase as the reporter through construction of a P*yvmC*-*lacZ* transcriptional fusion. The DNA carrying the promoter region was amplified by PCR using primers NSW2461 (5' -ATGCGAATTCTCATTAAGGTGCAGCAGTCTC-3') and NSW2482 (5'-GTATAAGCTTAATGCCTCAGCAATAAAATG-3') with genomic DNA extracted from NCIB 3610 being used as the template. The PCR fragment was digested using the EcoRI and HindIII restriction enzymes and ligated into pDG1728. The resulting plasmid pNW1728 was introduced into the genome of 168 and subsequently introduced into the genome of NCIB 3610.

Activity from the *dhbA* promoter was monitored using production of β-galactosidase as the reporter through construction of a P*dhbA*-*lacZ* transcriptional fusion. The DNA carrying the promoter region was amplified by PCR using primers NSW2477 (5' GTATGAATTCGCAGAATTTTGCGAGT-3') and NSW2478 (5' GTATGGTCCGTGCGCCTTGACTGGCA-3') with genomic DNA extracted from NCIB 3610 being used as the template. The PCR fragment was digested using the EcoRI and BamHI restriction enzymes and ligated into pDG1728. The resulting plasmid pNW1725 was introduced into the genome of 168 and subsequently introduced into the genomes of NCIB 3610, Δ*yvmC* (NRS5533) and Δ*cypX* (NRS5532) using SPP1 phage transduction.

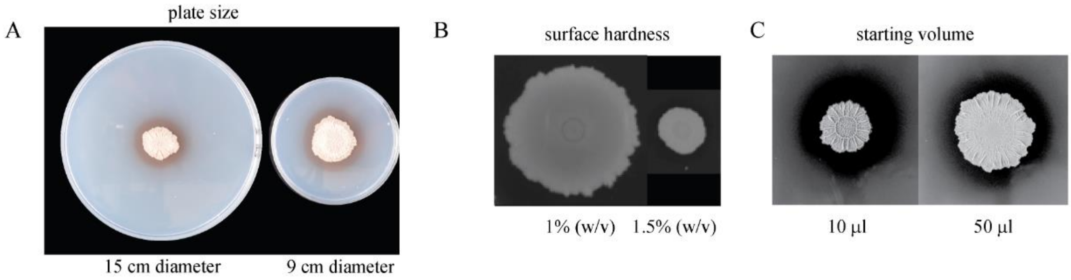

Fig. S1. **Environmental conditions influence the area colonised by the biofilm**. (**A**) NCIB 3610 biofilms formed on 15cm and 9 cm diameter plates; (**B**) NCIB 3610 biofilms formed on 9 cm diameter plates solidified with either 1% or 1.5% w/v agar; (**C**) NCIB 3610 biofilms formed on 9 cm diameter plates using different inoculation volume that contained the same number of cells.

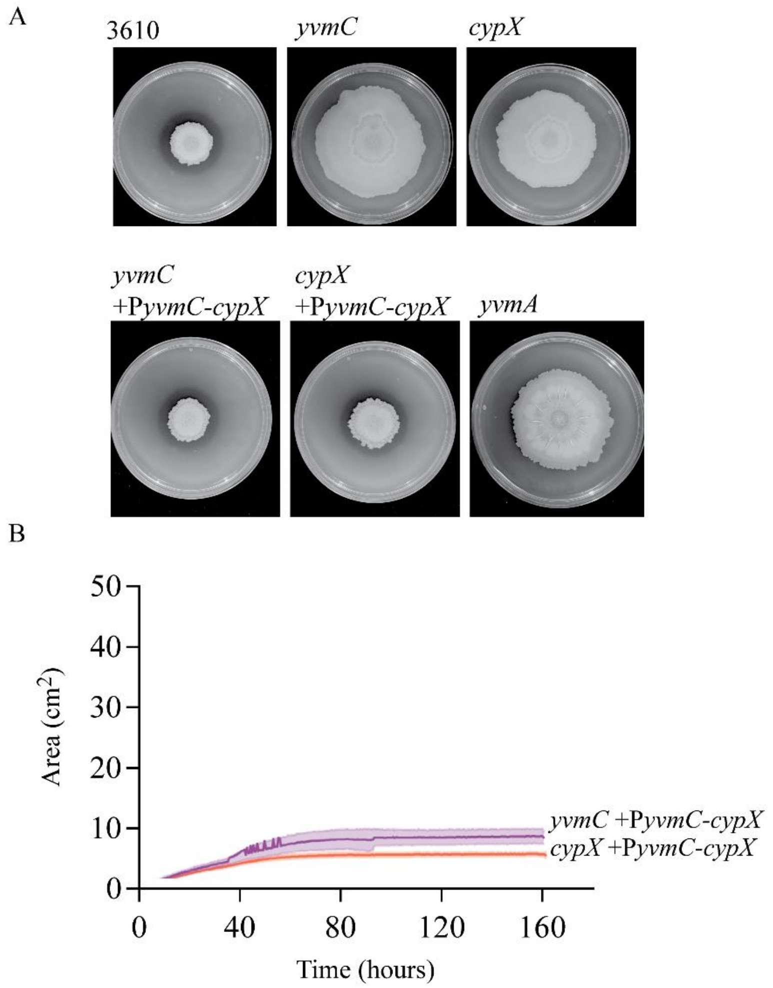

Fig. S2. Genetic complementation of the pulcherriminic acid deficient strains. (**A**) Representative images of biofilms formed by strains NCIB 3610, *cypX* (NRS5532), *yvmC* (NRS5533), *yvmC amyE:PyvmC-yvmC-cypX* (NRS6240), *cypX amyE:PyvmC-yvmC-cypX* (NRS6239) and *yvmA* (NRS6248) after 120 hours growth at 30°C on MSgg agar containing 50 μM $FeCl_3$.; (**B**) The area occupied by the biofilm formed by strains *yvmC amyE:PyvmC-yvmC-cypX* (NRS6240) and *cypX amyE:PyvmC-yvmC-cypX* (NRS6239) on the 9 cm diameter petri dish was calculated and plotted. The solid lines represent the average of 3 biological repeats and the shaded areas the standard error of the mean.

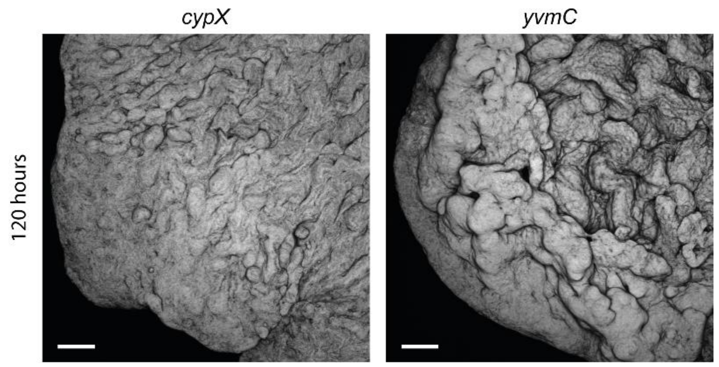

Fig. S3: Confocal analysis of the *cypX* and *yvmC* mutants at 120 hours. Confocal microscopy of the biofilm edge at 120 hours for the *cypX* (NRS5532) and *yvmC* (NRS5533) deletion strains. In each case the strains were mixed with an isogenic variant carrying a constitutively expressed copy of *gfp*. This allowed detection of a fraction of the cells in the biomass by confocal microscopy. The images shown are projections of the acquired z-stacks and the scale bars represent 100 μm.

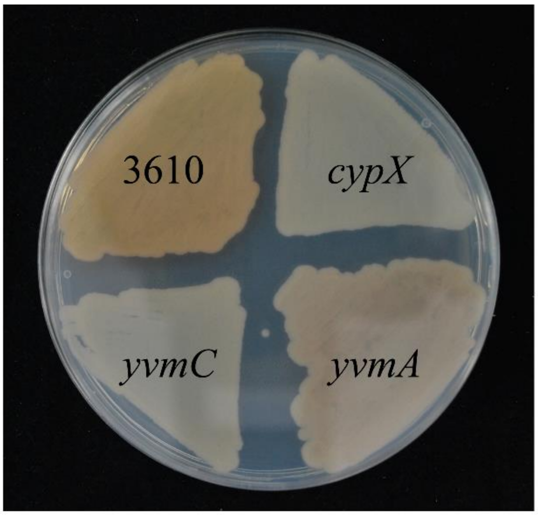

Fig. S4: YvmA transports pulcherriminic acid to the extracellular environment. NCIB 3610, *yvmC* (NRS5533), *cypX* (NRS5532) and *yvmA* (NRS6248) strains grown on an MSgg agar plate for 24 hours at 37°C prior to imaging.

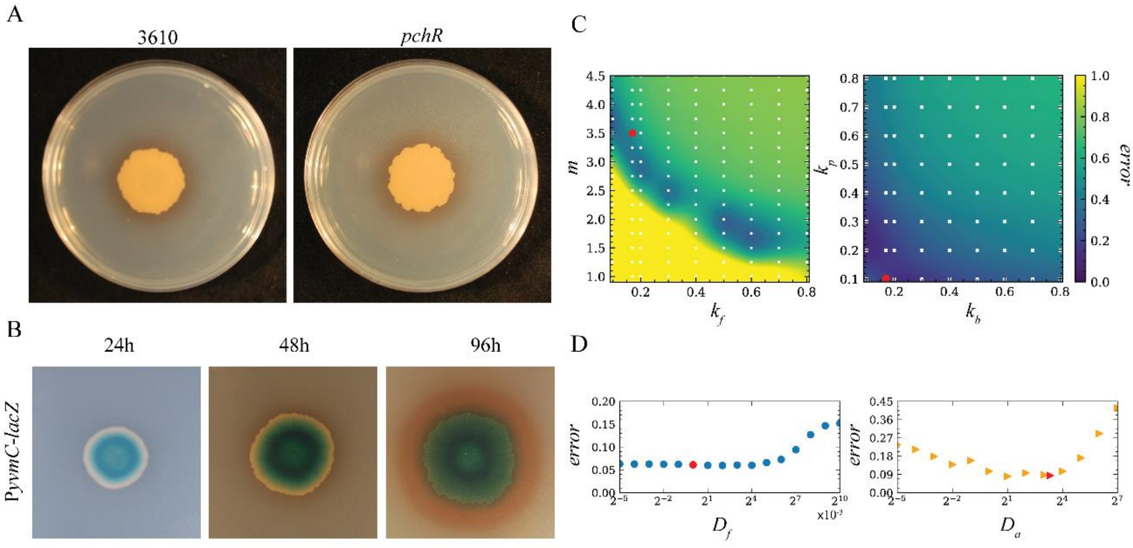

Fig. S5: Data supporting the parameters used in the mathematical model. (A) Photographs of 3610 and 3610 *pchR* (NRS6243) after growth at 30°C on MSgg agar for 48 hours. The image shown is representative of three biological repeats. **(B)** Images of 3610 containing the P*cypX-lacZ* transcriptional reporter fusion after growth at 30°C on MSgg agar in 9 cm diameter petri dishes containing 120 µg ml^{-1} 5-bromo-4-chloro-3-indolyl-β-D-galactopyranoside (X-gal) for 24, 48 and 96 hours. The image shown is representative of three biological repeats. **(C)** Model sensitivity analysis for (k_f, m) and (k_b, k_p) for fixed values of D_F, D_A and D_P as shown in Table S3. The colours show the relative error for the fitting procedure with dark blue regions representing low relative error and yellow high relative error zones, see colour bar. The white dots represent the values simulated. **(D)** For fixed values of parameters (see Table S3), the influence of iron (left) or pulcherriminic acid (right) diffusion coefficients on the relative error for the mutant (left) and wild type (right) experimental data were evaluated. For both (C) and (D) the red symbols represent the value shown in Table S3.

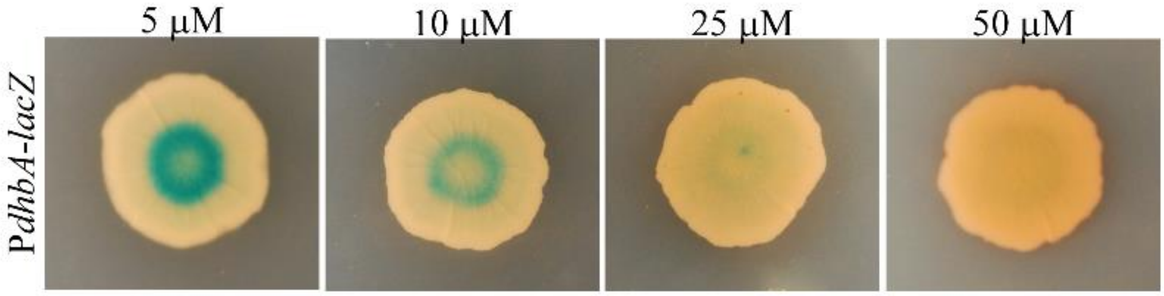

Fig. S6: P*dhbA* transcription monitored *in situ* during biofilm formation. Images of 3610 containing the P*dbh*A-*lacZ* transcriptional reporter fusion after growth at 30°C on MSgg agar in 9 cm diameter petri dishes containing 120 μg ml^{-1} 5-bromo-4-chloro-3-indolyl-β-D-galactopyranoside (X-gal) for 48 hours. The starting level of $FeCl_3$ in the growth medium was 5, 10, 25 and 50 μM as indicated. The image shown is representative of three biological repeats.

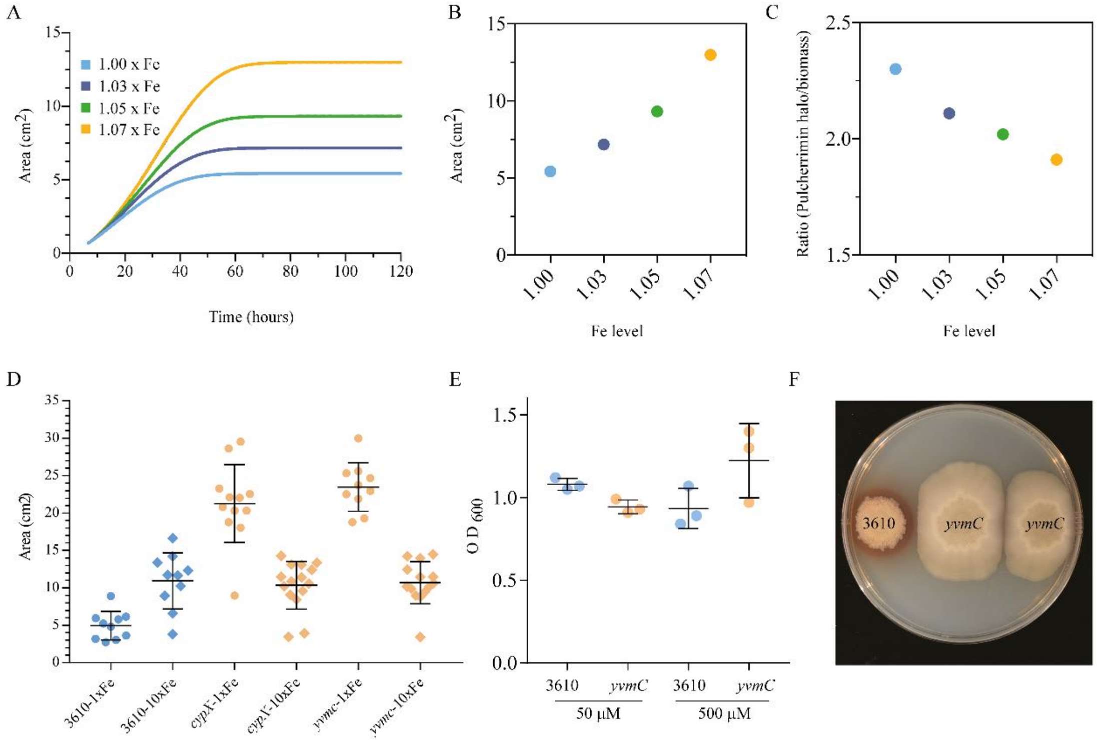

Fig. S7: Increasing the level of iron in the external environment transiently overcomes self-restriction of growth. (A) Model prediction for the biofilm surface area over time for different values of the initial iron concentration. **(B)** Terminal biofilm surface area for **(A)** versus the initial iron concentration. **(C)** Ratio of the Pulcherrimin halo radius to that of the biofilm footprint as given in **(B)**, versus the initial iron concentration. The boundary of the biomass is shown as a dotted line. **(D)** NCIB 3610 and the *cypX* (NRS5532) and *yvmC* (NRS5533) deletion strains were grown at 30°C on MSgg agar containing either 50 µM $FeCl_3$ (1x) or 500 µM $FeCl_3$ (10x) for 120 hours prior to calculation of the area occupied. The bar represents the average of the biological repeats that are presented as individual points, the error bars are the standard error of the mean; (**E**) NCIB 3610 and the *cypX* (NRS5532) and *yvmC* (NRS5533) deletion strains were grown for 10 hours in liquid MSgg medium containing either 50 µM $FeCl_3$ (1x) or 500 µM $FeCl_3$ (10x) prior to measurement of the cell density using OD_{600} measurements. The bar represents the average of the biological repeats that are presented as individual points, the error bars are the standard error of the mean; (**F**) Biofilms of NCIB 3610 and *yvmC* (NRS5533) were inoculated onto a 15 cm diameter MSgg agar (1.5% w/v agar) plate and incubated at 30°C for 120 hours prior to imaging.

Table S1. Full list of *B. subtilis* strains used in this study.

**Strain**	**Relevant genotype /Description** [a]	**Source / Construction** [b,c]
NCIB 3610	Prototroph	BGSC
168	*trpC2*	BGSC
BKE35060	168 *trpC2 cypX::erm*	BGSC (1)
BKE35070	168 *trpC2 yvmC::erm*	BGSC (1)
NRS5532	NCIB 3610 *cypX::erm*	SPP1 BKE35060 → NCIB3610
NRS5533	NCIB 3610 *yvmC::erm*	SPP1 BKE35070 → NCIB3610
BKK35080	168 *trpC2 pchR::kan*	BGSC (66)
NRS6243	NCIB 3610 *pchR::kan*	SPP1 BKK35080→ NCIB3610
NRS1473	NCIB 3610 *sacA::Pspachy-gfp::kan*	(2)
NRS5527	NCIB 3610 *cypX::erm sacA::Pspachy-gfp::kan*	SPP1 BKE35060 → NRS1473
NRS5529	NCIB 3610 *yvmC::erm sacA::Pspachy-gfp::kan*	SPP1 BKE35070 → NRS1473
DS1677	NCIB 3610 *Δhag*	Dan Kearns
NRS2097	NCIB 3610 *bslA::cml*	(3)
NRS2450	NCIB 3610 *eps(A-O)::tet*	(3)
NRS5267	NCIB 3610 *ΔtasA*	(4)
NRS5560	NCIB 3610 *Δhag yvmC::erm*	SPP1 BKE35070 → DS1677
NRS6281	NCIB 3610 *bslA::cml yvmC :: erm*	SPP1 BKE35070 → NRS2097
NRS6269	NCIB 3610 *eps(A-O)::tet yvmC::erm*	SPP1 BKE35070 → NRS2450
NRS6276	NCIB 3610 *ΔtasA yvmC::erm*	SPP1 BKE35070 → NRS5267
BKK35090	168 *trpC2 yvmA::kan*	BGSC (1)
NRS6248	NCIB 3610 *yvmA::kan*	SPP1 BKK35090 → NCIB3610
NRS6238	168 *trpC2 amyE::PyvmC$_{500bp}$-yvmC-cypX::spc*	pNW1722 → 168
NRS6239	NCIB 3610 *cypX::erm amyE::PyvmC$_{500bp}$-yvmC-cypX::spc*	SPP1 NRS6238 → NRS5532
NSW6275	168 *trpC2 amyE::PyvmC-lacZ::spc*	pNW1728 → 168
NSW6279	NCIB 3610 *amyE::PyvmC-lacZ::spc*	SPP1 NRS6275 → NCIB3610
NRS6240	NCIB 3610 *yvmC::erm amyE::PyvmC$_{500bp}$-yvmC-cypX::spc*	SPP1 NRS6238 → NRS5533
NRS6256	168 *trpC2 amyE::PdhbA-lacZ::spc*	pNW1725 → 168
NRS6257	NCIB 3610 *amyE::PdhbA-lacZ::spc*	SPP1 NRS6256 → NCIB3610
NRS6258	NCIB 3610 *cypX::erm amyE::PdhbA-lacZ::spc*	SPP1 NRS6256 → NRS5532
NRS6259	NCIB 3610 *yvmC::erm amyE::PdhbA-lacZ::spc*	SPP1 NRS6256 → NRS5533

a. Drug resistance cassettes are indicated as follows: *cml*, chloramphenicol resistance; kan, kanamycin resistance; *erm*, erythromycin resistance; *neo*, neomycin resistance; *tet*, tetracycline

resistance and *spc*, spectinomycin resistance. BSGC represents the *Bacillus* genetic stock centre.

b. The direction of strain construction is indicated with DNA or phage (SPP1) (→) recipient strain. The reference is provided if the strain has previously been described.

Table S2. : Details of strains used for phylogenetic tree construction.

Species/strain identified by 16S analysis	Accession number	Database classification	Source
Bacillus subtilis subsp. subtilis NCIB 3610	CP020102.1	*Bacillus subtilis subsp. subtilis NCIB 3610*	ENA Release 137
Aeribacillus pallidus	GCA_001624605.1	*Geobacillus sp 8*	Ensembl Genomes release 40
Bacillus albus	GCA_000238655.1	*Bacillus sp 7 6 55cfaa ct2*	Ensembl Genomes release 40
Bacillus amyloliquefaciens	GCA_000696285.1	*Bacillus amyloliquefaciens*	Ensembl Genomes release 40
Bacillus anthracis	GCA_000725325.1	*Bacillus anthracis*	Ensembl Genomes release 40
Bacillus bombysepticus	GCA_000831065.1	*Bacillus bombysepticus*	Ensembl Genomes release 40
Bacillus cereus	GCA_000160915.1	*Bacillus cereus*	Ensembl Genomes release 40
Bacillus licheniformis	GCA_000952085.1	*Bacillus licheniformis*	Ensembl Genomes release 40
Bacillus mobilis	GCA_001429465.1	Bacillus sp root11	Ensembl Genomes release 40
Bacillus murimartini	GCA_001274705.1	*Bacillus murimartini*	Ensembl Genomes release 40
Bacillus paralicheniformis	GCA_000746885.1	*Bacillus paralicheniformis*	Ensembl Genomes release 40
Bacillus pumilus	GCA_000590455.1	*Bacillus pumilus*	Ensembl Genomes release 40
Bacillus subtilis subsp. inaquosorum	GCA_000332645.1	*Bacillus subtilis subsp. inaquosorum*	Ensembl Genomes release 40
Bacillus subtilis subsp spizizenii	GCA_000816805.1	*Bacillus subtilis subsp spizizenii*	Ensembl Genomes release 40
Bacillus tequilensis	GCA_001278955.1	*Bacillus tequilensis*	Ensembl Genomes release 40
Bacillus thuringiensis	GCA_000600315.1	*Bacillus thuringiensis*	Ensembl Genomes release 40
Clostridium perfringens	CP019468.1	*Clostridium perfringens*	ENA Release 137
Geobacillus icigianus	GCA_001587475.1	*Geobacillus sp b4113 201601*	Ensembl Genomes release 40
Geobacillus thermodenitrificans	GCA_001587475.1	*Geobacillus thermodenitrificans*	Ensembl Genomes release 40
Jeotgalibacillus marinus	GCA_001274925.1	*Jeotgalibacillus marinus*	Ensembl Genomes release 40
Listeria monocytogenes	GCA_000382925.1	*Listeria monocytogenes*	Ensembl Genomes release 40
Staphylococcus aureus	GCA_000597965.1	*Staphylococcus aureus*	Ensembl Genomes release 40
Staphylococcus delphini	PRJEA8701	*Staphylococcus delphini*	ENA Release 137
Staphylococcus epidermidis	GCA_000759555.1	*Staphylococcus epidermidis*	Ensembl Genomes release 40
Staphylococcus equorum	GCA_001747895.1	*Staphylococcus equorum*	Ensembl Genomes release 40
Staphylococcus haemolyticus	GCA_000009865.1	*Staphylococcus haemolyticus*	Ensembl Genomes release 40
Staphylococcus lugdunensis	GCA_000247225.2	*Staphylococcus lugdunensis*	Ensembl Genomes release 40
Staphylococcus pseudintermedius	GCA_001622965.1	*Staphylococcus pseudintermedius*	Ensembl Genomes release 40

Table S3: Non-dimensional system parameters and their values

Descriptor	Symbol	Non-Dimensional Value
Biomass density (assumed constant within its footprint)	B	1
Initial iron concentration in the agar	F_0	1*
Iron level resulting in half maximal growth rate	F_h	1
Biomass material constant	λ	1
Growth response to change in iron level	m	3.5
Rate constant for pulcherriminic acid production wild type	k_b	0.17
Rate constant for pulcherriminic acid minus strains	k_b	0
Rate constant for iron chelation (pulcherrimin production)	k_p	0.6 k_b
Rate constant for iron utilisation due to biomass production	k_f	0.17
Pulcherriminic acid diffusion rate constant	D_A	10
Iron diffusion rate constant	D_F	10^{-4} D_A
Pulcherrimin diffusion rate constant	D_P	10^{-4} D_A

*The value of initial free iron was varied as illustrated in Figure S7.

References

1. Koo BM, *et al.* (2017) Construction and Analysis of Two Genome-Scale Deletion Libraries for *Bacillus subtilis*. *Cell systems* 4(3):291-305 e297.
2. Verhamme DT, Kiley TB, & Stanley-Wall NR (2007) DegU co-ordinates multicellular behaviour exhibited by *Bacillus subtilis*. *Mol Microbiol* 65(2):554-568.
3. Ostrowski A, Mehert A, Prescott A, Kiley TB, & Stanley-Wall NR (2011) YuaB functions synergistically with the exopolysaccharide and TasA amyloid fibers to allow biofilm formation by *Bacillus subtilis*. *J Bacteriol* 193(18):4821-4831.
4. Erskine E, *et al.* (2018) Formation of functional, non-amyloidogenic fibres by recombinant Bacillus subtilis TasA. *Molecular microbiology*.
